# Arteriovenous Malformation in the Auricle in a 59-Year-Old Woman

**DOI:** 10.1155/2021/7438571

**Published:** 2021-10-08

**Authors:** Junhui Jeong

**Affiliations:** Department of Otorhinolaryngology, National Health Insurance Service Ilsan Hospital, Goyang, Republic of Korea

## Abstract

Arteriovenous malformation (AVM) is a vascular lesion with a direct communication between an artery and a vein without a capillary system. AVM primarily occurs in the intracranial area, but can also occur in the extracranial area. If there is a tender mass with pulsation or recurrent bleeding in the auricle, AVM should be considered even though it rarely occurs in the auricle. AVM in the ear should be managed carefully because the skin is thin in the ear, the cartilage could be involved, and progressive growth or inappropriate management could cause bleeding, infection, and cosmetic problems such as deformity. I present a case of a 59-year-old woman with AVM in the auricle.

## 1. Introduction

Arteriovenous malformation (AVM) is a vascular lesion with a direct communication between an artery and a vein without a capillary system. Mulliken and Glowacki classified vascular lesions into hemangiomas and vascular malformations [1–3]. A hemangioma is comprised of increased numbers of vessels filled with blood [3], whereas AVM consists of vascular channels with flat mature endothelium [1]. Increased endothelial cell turnover differentiates a hemangioma from AVM [4]. An AVM is almost always present at birth; however, it manifests later in life [5]. An AVM usually presents during childhood, however, it may remain quiescent until adolescence or even into adulthood [4, 5]. AVM may be enlarged by trauma, infection, or hormonal influences [1, 4, 5].

AVM primarily occurs in the intracranial area, but can also occur in the extracranial area. Extracranial AVM are most frequently identified in the head and neck region; the most common site is the cheek followed by the ear [5, 6]. AVM in the ear should be managed carefully because the skin is thin in the ear, the cartilage could be involved, and progressive growth or inappropriate management could cause bleeding, infection, and cosmetic problems such as deformity [7]. I present a case of a 59-year-old woman with AVM in the auricle.

## 2. Case Presentation

**Figure 1:**
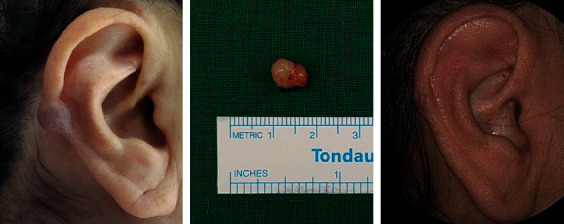
A soft and cystic mass with purple skin discoloration in the helix of the right auricle (a). Surgical specimen of the 0.8 × 0.6 cm, whitish, soft, cystic mass (b). Postoperative wound which healed well after 2 months (c).

**Figure 2:**
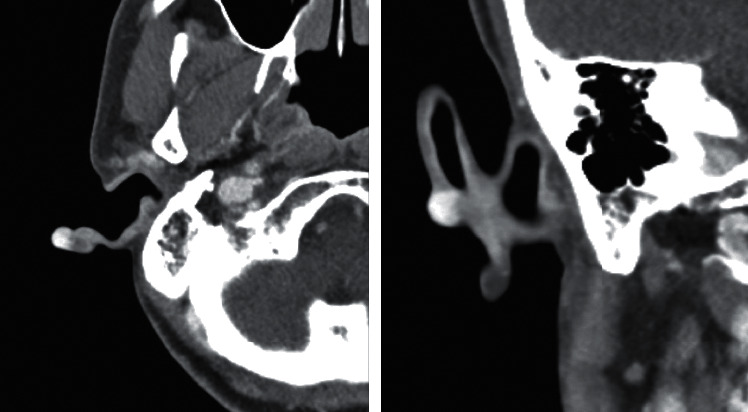
Facial computed tomography revealing a 0.8 × 0.6 cm strongly enhanced mass in the right auricle. (a) Axial image; (b) coronal image.

A 59-year-old woman visited my otorhinolaryngology clinic for a cystic mass which had caused intermittent pain in the right auricle for several years. She had no history of trauma or infection in that area. On physical examination, a soft and cystic mass with purple skin discoloration was observed in the helix of the right auricle (Figure 1(a)). Facial computed tomography revealed a 0.8 × 0.6 cm, strongly enhanced mass in the right auricle (Figure 2). Initial evaluation suspected a vascular tumor such as a hemangioma; therefore, surgical excision was planned because the patient wanted the mass removed.

Under local anesthesia, the skin was incised, and the whitish soft cystic mass was dissected and removed (Figure 1(b)). The mass did not involve the conchal cartilage. Histopathologic findings revealed AVM. The wound healed well after 2 months (Figure 1(c)), and there was no recurrence after 6 months.

## 3. Discussion

The pathogenesis of AVM has not been elucidated [1, 2]. It is hypothesized that AVM is due to failed regression of arteriovenous channels in the primitive retiform plexus during the 4th to 6th gestation weeks of fetal development. Local ischemia is also thought to play a role; thus, AVM expands after proximal ligation. Trauma may enlarge the AVM in this respect [2].

Schobinger classified AVM into four stages. In stage I (quiescence), the symptom is warm and discolored skin; in stage II (expansion), the symptoms are bruit, pulsation, and swelling. In stage III (destruction), there is pain, ulceration, and bleeding; and stage IV (decompensation) is characterized by cardiac failure [5]. Based on the flow rate, there are two types of AVM, which are fast flowing and slow flowing. Most fast-flowing lesions are arteriovenous fistulas whereas slow-flowing lesions are venous, capillary, or lymphatic lesions [1, 5]. Treatment approaches may differ between these two types [5].

Plain X-ray and computed tomography have limitations for evaluating AVM. Magnetic resonance imaging and magnetic resonance angiography are the best approaches for evaluating the extension and confirming the diagnosis. In angiography, AVM shows tortuous and dilated arteries with arteriovenous shunts and dilated draining veins [2].

The most common symptoms of AVM in the auricle are pulsation, bleeding, and pain. Treatment is not necessary if the AVM is small and does not induce symptoms [5]. Treatment approaches can include surgery, embolization, sclerotherapy, and radiotherapy or a combination of these approaches. Treatment selection is determined by size, location, angioarchitecture of the mass, and adjacent structures such as the skin [6]. Complete excision preceded by embolization is the treatment of choice for symptomatic AVM cases [4, 5]. The purpose of treatment is not only an angiographic cure but also a cosmetically favorable result and resolution of symptoms [6]. AVM in the auricle may be associated with swelling and deformity; thus, it can be misdiagnosed as an otohematoma. Drainage based on this misdiagnosis may induce massive bleeding [5].

In the auricle, blood is supplied by superficial temporal, posterior auricular, and occipital arteries. The nidus of the AVM is usually supplied by one or more of these main vessels [7]. With embolization, nidus and venous outflow are occluded [2]. Surgical ligation or embolization should not be proximal to prevent aggravation by new collaterals [4]. Surgical excision has a lower recurrence than embolization alone [2]. Surgery should be performed within 48 hours after embolization [1, 4].

The incidence of AVM in the auricle is low, and there are limited reports on the guidelines for managing it. Vilela Chagas Ferreira et al. reported a protocol for resection and reconstruction of the AVM in the auricle. They recommended total resection with cartilage preservation for the lesion without cartilaginous involvement; total resection with cartilage resection was recommended for partial involvement of the auricle with cartilaginous involvement; auricular amputation was recommended for total involvement of the auricle or extra-auricular involvement with cartilaginous involvement. Preoperative embolization was not recommended for partial involvement of the auricle without cartilaginous involvement. Consequently, immediate or delayed reconstruction might be necessary [7].

If there is a tender mass with pulsation or recurrent bleeding in the auricle, AVM should be considered even though it rarely occurs in the auricle. For a symptomatic large-sized and cartilage-involved AVM, superselective embolization followed by surgical excision is necessary.

## Data Availability

No data were used to support this study.
